# Cross-modal associations of language brain areas in relation to auditory hallucinations in schizophrenia

**DOI:** 10.1192/j.eurpsy.2025.2263

**Published:** 2025-08-26

**Authors:** P. Salgado-Pineda, L. Barbosa, J. Soler-Vidal, N. Ramiro, P. Fuentes-Claramonte, P. J. McKenna, E. Pomarol-Clotet, P. del Olmo-Encabo

**Affiliations:** 1 FIDMAG- GermanesHospitalàries Research Foundation; 2 CIBERSAM-ISCIII; 3 FIDMAG Germanes Hospitalàries Research Foundation, Barcelona, Spain

## Abstract

**Introduction:**

An influential current theory suggests that auditory verbal hallucinations (AVH) result from abnormal activity in the auditory cortex. Against this, however, Fuentes-Claramonte *et al* (Sci Rep 2021; 11 18890) recently found that AVH did not activate the primary or secondary auditory cortex, although there were activations in other areas including Broca’s area. At the level of brain structure, sulcal depth has been increasingly linked to brain function (Natu *et al* Cereb Cortex 2021; 31 48-61; De Vareilles *et al* Dev Cogn Neurosci 2023; 61 101249 ). Accordingly, exploring brain structural-functional associations for AVH may provide new insights in their biological basis.

**Objectives:**

To assess the relationship between sulcal depth and brain activity during AVH and perception of real speech.

**Methods:**

Functional (fMRI) and structural (sMRI) 3T scans were obtained from 14 patients with schizophrenia who experienced near-continuous AVH. During fMRI, participants pressed a button when they experienced AVH or heard real speech similar in form to their AVH. Standard fMRI analysis was conducted with FSL, while sMRI images were processed using Freesurfer’s recon-all pipeline to measure sulcal depth. Cross-modal registration aligned whole-brain fMRI activation maps to corresponding structural data and correlations between sulcal depth and brain activity were calculated for each vertex; age, sex and estimated premorbid IQ were covaried for. Cluster-based correction was applied for multiple comparisons.

**Results:**

During real speech, a positive correlation was found between brain activations and sulcal depth in the left superior temporal sulcus (STS, BA 22), and negative correlations in the middle temporal (BA 21), frontal (BA 46), and parietal cortex. On the right, positive correlations were seen in the superior and middle temporal cortex (BA 38, 20, 42), while negative correlations were found in the STS (BA 22), pars triangularis (BA45), and precentral (figure a). During AVH, there was a negative correlation in the left pars triangularis (BA 45) only, including Broca’s area, with no significant correlations in the right hemisphere (figure b).

**Image 1:**

**Figure:**
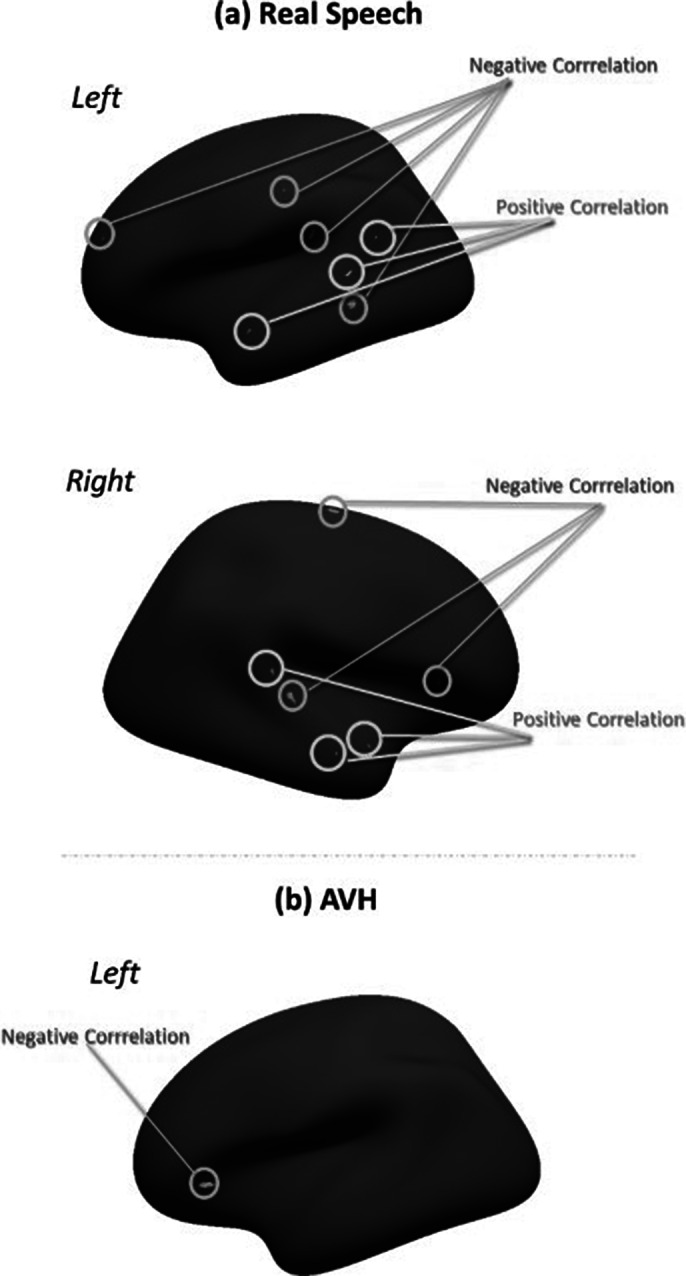

**Conclusions:**

The left STS, along with frontal and temporoparietal areas, appear structurally and functionally linked to perception of real speech. In contrast, AVH primarily engages Broca’s area and adjacent left inferior frontal regions.

**Disclosure of Interest:**

None Declared

